# CircBCAR3 accelerates esophageal cancer tumorigenesis and metastasis via sponging miR-27a-3p

**DOI:** 10.1186/s12943-022-01615-8

**Published:** 2022-07-15

**Authors:** Yong Xi, Yaxing Shen, Donglei Wu, Jingtao Zhang, Chengbin Lin, Lijie Wang, Chaoqun Yu, Bentong Yu, Weiyu Shen

**Affiliations:** 1 Department of Thoracic Surgery, Ningbo Medical Center Lihuili Hospital, Ningbo University, Ningbo, 315040 Zhejiang China; 2 Department of Thoracic Surgery, Zhongshan Hospital, Fudan University, Shanghai, 20032 China; 3 School of Medicine, Jinan University, Guangzhou, 510627 Guangdong China; 4 Department of Thoracic Surgery, The First Affiliated Hospital of Nanchang University, Nanchang, 330006 Jiangxi China

**Keywords:** hsa_circ_0007624, Esophageal cancer, Hypoxia, Splicing factor quaking, Ferroptosis

## Abstract

**Rationale:**

Circular RNAs (circRNAs) have been demonstrated to contribute to esophageal cancer progression. CircBCAR3 (hsa_circ_0007624) is predicted to be differentially expressed in esophageal cancer by bioinformatics analysis. We investigated the oncogenic roles and biogenesis of circBCAR3 in esophageal carcinogenesis.

**Methods:**

Functions of circBCAR3 on cancer cell proliferation, migration, invasion, and ferroptosis were explored using the loss-of-function assays. A xenograft mouse model was used to reveal effects of circBCAR3 on xenograft growth and lung metastasis. The upstream and downstream mechanisms of circBCAR3 were investigated by bioinformatics analysis and confirmed by RNA immunoprecipitation and luciferase reporter assays. The dysregulated genes in hypoxia-induced esophageal cancer cells were identified using RNA-seq.

**Results:**

CircBCAR3 was highly expressed in esophageal cancer tissues and cells and its expression was increased by hypoxia in vitro. Silencing of circBCAR3 repressed the proliferation, migration, invasion, and ferroptosis of esophageal cancer cells in vitro, as well as inhibited the growth and metastasis of esophageal xenograft in mice in vivo. The hypoxia-induced promotive effects on esophageal cancer cell migration and ferroptosis were rescued by circBCAR3 knockdown. Mechanistically, circBCAR3 can interact with miR-27a-3p by the competitive endogenous RNA mechanism to upregulate transportin-1 (TNPO1). Furthermore, our investigation indicated that splicing factor quaking (QKI) is a positive regulator of circBCAR3 via targeting the introns flanking the hsa_circ_0007624-formed exons in BCAR3 pre-mRNA. Hypoxia upregulates E2F7 to transcriptionally activate QKI.

**Conclusion:**

Our research demonstrated that splicing factor QKI promotes circBCAR3 biogenesis, which accelerates esophageal cancer tumorigenesis via binding with miR-27a-3p to upregulate TNPO1. These data suggested circBCAR3 as a potential target in the treatment of esophageal cancer.

**Graphical Abstract:**

Hypoxia induces the upregulation of E2F7, which transcriptionally activates QKI in esophageal cancer cells. QKI increases the formation of circBCAR3 by juxtaposing the circularized exons. CircBCAR3 binds with miR-27a-3p to promote TNPO1 expression. CircBCAR3 promoted the proliferation, migration, invasion, and ferroptosis of esophageal cancer cells by miR-27a-3p.
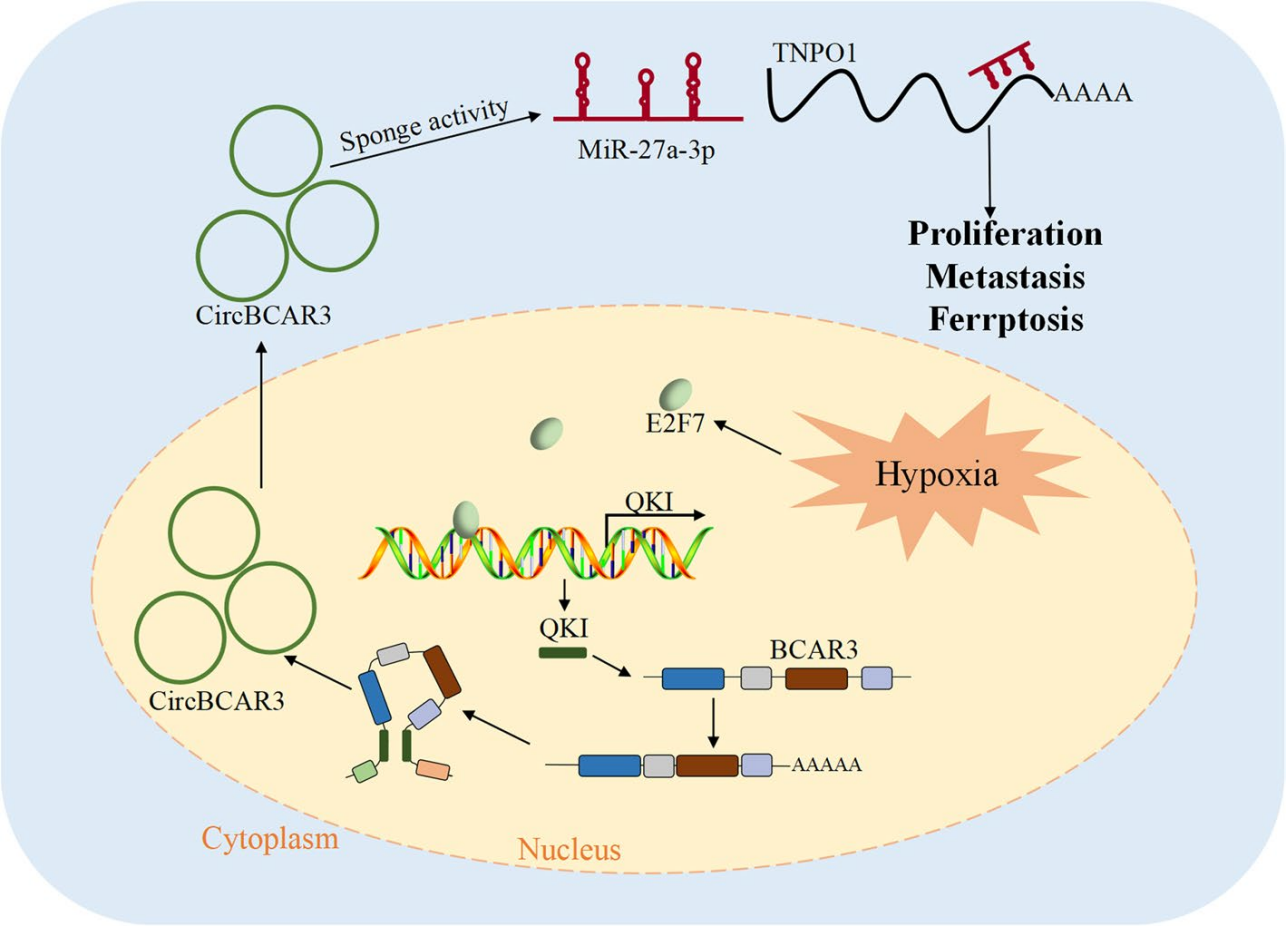

**Supplementary Information:**

The online version contains supplementary material available at 10.1186/s12943-022-01615-8.

## Introduction

Esophageal cancer is a globally fatal cancer with an overall 5-year survival rate of 18% [1]. The incidence, mortality, and histopathology of esophageal cancer are different in different geographic regions, particularly causing high burden in residents in Eastern/Southern Africa and East Asia [2]. Adenocarcinoma and squamous cell carcinoma are the two predominant histological subtypes that account for more than 95% of esophageal cancers [3, 4]. There are some advances in the therapeutic methods of this cancer in the past decades. At present, it is needed to develop new treatments targeting the oncogenesis of esophageal cancer.

Noncoding RNAs (ncRNAs) including long ncRNAs, microRNAs (miRNAs), tRNA-derived small RNAs, piwi-interacting RNAs, and pseudogenes play important regulatory roles in tumorigenesis and progression of all human tumors. Recently, circular RNAs (circRNAs), as an emerging new subgroup of endogenous ncRNAs, can function as crucial regulators in cell cycle, apoptosis, proliferation, invasion, and migration in cancers [5–7]. They are generated by the back splicing of pre-mRNAs to form a covalently closed loop without a 5′-cap or a 3′-poly(A) tail [8], and have the potential to be diagnostic and prognostic biomarkers for cancers because of their widespread expression, relatively high stability, and abundant presence in saliva, blood, and exosomes [9]. CircRNAs can act as miRNA sponges to regulate the pathogenesis of tumors [10, 11] including squamous cell carcinoma [12]. Most of the studies focused on the biological roles of circRNAs in the normal condition. The biogenesis and function of circRNAs in the low oxygen condition remain unclear. Hypoxia can destroy normal tissue homeostasis and rearrange tumor matrix interaction. Under the condition of hypoxia, tumor cells become more likely to migrate. Previous studies have reported that hypoxia can induce tumor metastasis [13, 14]. Clinically, tumor hypoxia is associated with high resistance to radiation therapy or chemotherapy and poor prognosis [15, 16]. Thus, exploring the circRNAs in esophageal cancer under the hypoxia condition has significant meanings.

Ferroptosis is a metabolic stress-induced cell death caused by cystine depletion and overproduction of lipid reactive oxygen species (ROS) in an iron-dependent manner [17, 18] and is involved in ischemic damage and cancer [19]. Glutathione peroxidase 4 (GPX4) reduces lipid hydroperoxides to lipid alcohols by reducing glutathione (GSH), thus protecting cells against membrane lipid peroxidation and inhibiting ferroptosis [20, 21]. Morphological features of ferroptosis include decreased mitochondrial volume, reduced or absent mitochondrial crest, ruptured mitochondrial outer membrane, a normal-sized nucleus, and no nuclear concentration, which distinguishes it from other modes of death [18]. Since cancer and normal cells show physiological differences in iron and lipid metabolism along with aberrant ROS production [22, 23], cancer cells may be more susceptible to modulation in ferroptosis than normal cells. Approved drugs including sulfasalazine, sorafenib, artemisinin, and statins can induce ferroptosis and suppress tumor growth [24]. However, ferroptosis, as a double-edged sword, can cause immunosuppression in tumor microenvironment and thus contributes to tumor growth [24]. A previous study revealed that migration-prone cancer cells are highly sensitive to ferroptosis [25]. Another study revealed that hypoxia can protect tumor cells from ferroptosis [26]. The present study revealed that ferroptosis is activated by hypoxia in esophageal cancer cells and investigated the role of hypoxia-induced circRNA in ferroptosis and its underlying mechanism.

Generations of circRNAs via back-splicing reactions are combinatorically controlled by exon skipping events and RNA binding proteins (RBPs) [27]. RBPs, including ESRP1, ESRP2, PTBP1, TNPO1, RBM, as well as QKI, can perform the functions of splicing factors to regulate the alternative splicing [28]. Quaking (QKI), belonging to the STAR family of KH domain-containing RNA binding proteins, can affect pre-mRNA splicing [29]. The introduction of consensus binding sequences for QKI into the flanking introns causes the generation of circRNAs from exons that only go through conventional linear splicing in normal circumstances [30]. Since QKI can form dimers, it was deemed to target flanking introns and bring the circularized exons closer together, leading to the increased generation of circRNAs [31].

In the study, we reported that circBCAR3 (hsa_circ_0007624), a novel identified circRNA in esophageal cancer cells, is generated from the 2, 3, 4, 5 exons of BCAR3 pre-mRNA with genomic location of chr1:94047857–94,057,950. We further demonstrated that circular RNA circBCAR3 was upregulated in the esophageal cancer tissues and cells and regulated the proliferation, migration, invasion, and ferroptosis of esophageal cancer cells in vitro. CircBCAR3 expression was increased by hypoxia and rescued the effects of hypoxia on esophageal cancer cells. Silenced circBCAR3 inhibited the esophageal cancer tumor growth and metastasis in vivo. Mechanistically, E2F7-induced splicing factor QKI increased circBCAR3.

## Materials and methods

### Bioinformatics analysis

The GSE150476 and GSE112496 datasets were used to reveal the differentially expressed circRNAs in esophageal cancer. Five circRNAs including hsa_circRNA_103225, hsa_circRNA_404013, hsa_circRNA_102471, hsa_circRNA_007624 (also termed hsa_circ_0007624 and was abbreviated as circBCAR3 in the present study), and hsa_circRNA_082546 were found to be differentially expressed in esophageal cancer based on the intersections of GSE150476 and GSE112496. The ENCORI database [32] was used to reveal the miRNAs binding to hsa_circ_0007624 and the mRNAs binding with miR-27a-3p. Under the condition of strict stringency (> = 5) of CLIP Data, miR-27a-3p was found to rank first as the target of hsa_circ_0007624. Under the condition of strict stringency (> = 5) of CLIP Data, high stringency (> = 3) of Degradome Data, more than 10 cancer types in Pan-Cancer, and more than 5 predicted programs, TNPO1, GCC2, THRB, CASC3 were found as the targets of miR-27a-3p. The binding sites of hsa_circ_0007624/TNPO1 and miR-27a-3p as well as the motif of QKI were also obtained from ENCORI. The GEPIA database [33] was used to predict the expression profile of TNPO1 and E2F7 in esophageal cancer tissues and the expression correlation between TNPO1 and QKI/E2F7. Metascape [34] was used for GO enrichment analysis of the hypoxia-induced differentially expressed RNAs in EC109 cells. Motif of E2F7 was obtained from the JASPAR database [35].

### Tissue samples

Forty-five paired esophageal cancerous and adjacent noncancerous specimens (at least 5 cm away from the tumor tissues) were collected from patients by surgical operation at Ningbo Medical Center Lihuili Hospital, Ningbo University. The esophageal cancerous tissues were confirmed by histological examination. All participants had signed the written informed consents before the study. Only resected samples from patients underwent surgery with written informed consent were included. These tissue samples were immediately snap-frozen in liquid nitrogen and subsequently stored at − 80 °C. This study was approved by the Ethics Committees of Ningbo Medical Center Lihuili Hospital, Ningbo University and was performed in accordance with the principles of Declaration of Helsinki.

### Cell culture

Human esophageal carcinoma cell lines EC109 (#MZ-2019, well differentiated squamous carcinoma, MINGZHOU BIO, Ningbo, China), KYSE30 (#ACC-351, well differentiated squamous carcinoma, DSMZ, Germany), KYSE70 (#ACC-379, poorly differentiated squamous carcinoma, DSMZ), KYSE150 (#ACC-375, poorly differentiated squamous carcinoma, DSMZ), KYSE180 (#ACC-379, well differentiated squamous carcinoma, DSMZ), KYSE450 (#ACC-387, well differentiated squamous carcinoma, DSMZ), and normal human esophagus epithelial cell line Het-1A (#BFN60806666, BLUEFBIO, China) were used. The cells were cultured in RPMI-1640 with 1% penicillin/streptomycin (#PM150110A, Procell, Wuhan, China) at 37 °C/5% CO_2_. 10% FBS (#10093, ThermoFisher) was added into the culture medium. Cells were tested for mycoplasma contamination by PCR method twice a month.

### Cell transfection

CircBCAR3 overexpressing plasmid (pcDNA circBCAR3), silencing plasmid (sh-circBCAR3), and their negative controls (empty pcDNA and sh-NC) were commercially provided by GenePharma (Shanghai, China). The miR-27a-3p mimic/inhibitor and their negative controls were purchased from Ribobio Biotech (Guangzhou, China). The abovementioned plasmids and negative controls were transfected into EC109 and KYSE150 cells using Lipofectamine RNAiMax (#13778150, Life Technologies). Samples were harvested after 24 h of transfection for the further research.

### Quantitative real-time polymerase chain reaction

TRIzol (#15596026, Thermo Fisher Scientific, MA, USA) was used for extraction of total RNA from cancer tissues and cells. Reverse transcription of RNAs was performed using PrimeScript RT Master Mix (Takara, Dalian, China) based on the manufacturer’s protocols. The cDNA was amplified using SYBR Premix Ex Taq (#RR420A; Takara). qRT-PCR was then conducted on an Applied Biosystems™ 7500 Fast Real-Time PCR System (#4351106). The cDNA and gDNA PCR products were observed with 2% agarose gel electrophoresis. The expression of circRNAs and mRNAs was normalized to GAPDH, while expression of miRNAs was normalized to U6. Gene expression were calculated by the 2^−ΔΔCt^ method [36]. The primer sequences were listed as following: circBCAR3, F: 5′-CCTGGAAACAGCAATGTTGA-3′; R: 5′-GTCCATGATGTGCCTCTCCT-3′. TNPO1, F: 5′-GTCTTAACAGAGTTAGAACTTGGG-3′; R: 5′-CTTCTGGGAGTATCTTGAAAGAG-3′. QKI, F: 5′-ATTATTGGTACCTGCAGCAG-3′; R: 5′-TAGGTGCCATTCAGAATCG-3′. E2F7, F: 5′-CTCGCTATCCAAGTTATCCC-3′; R: 5′-TTTCCACACCAAGACTGAC-3′. GAPDH, F: 5′-TCAAGATCATCAGCAATGCC-3′; R: 5′-CGATACCAAAGTTGTCATGGA-3′.

### Treatment of RNase R and actinomycin D

Two micrograms of total RNA was cultured with 5 U/μg RNase R (#ab286929, Abcam) for half an hour at 37 °C. EC109 and KYSE150 cells were treated with 2 μg/mL actinomycin D (#ab291108, Abcam) for 1, 2, 4, 8, 12 h. RNA expression of circBCAR3 and linear BCAR3 was analyzed using qRT-PCR.

### Western blot analysis

Proteins were extracted by RIPA lysis buffer (P0013B, Beyotime), separated on 8% SDS-PAGE, transferred to a PVDF membrane (IPVH00010, Millipore, USA), and blocked with 3% skim milk powder at 37 °C for 60 min. The PVDF membrane were incubated with primary antibodies at 4 °C overnight and then incubated with appropriate HRP-labelled secondary antibodies. A ECL System (WBULS0500, Millipore) was used to develop the films. The primary antibodies against GPX4 (#52455, 1:2000) and GAPDH (#3316 s, 1:2000) were purchased from Cell Signaling Technology (Danvers, MA, USA).

### Luciferase reporter assay

Dual-luciferase reporter vectors carrying the wild type (WT) fragments (5′- UAUUUUCUUAUAUACUGUGAA-3′) of TNPO1 3’untranslated region (3’UTR) or the mutant (MUT) fragments (5′-UAUUUUAGGCUAUGCACGACA-3′) were constructed and termed TNPO1-wt or TNPO1-mut. Similarly, the wild fragments (5′-TTTTCCCGCCACCT-3′) of QKI promoter that were complementary to E2F7 or the mutant fragments (5′-TCATCGTGCACCGT-3′) were subcloned into the pGL3 vector to construct the QKI-wt or QKI-mut vectors. To assess the binding between miR-27a-3p and TNPO1 3’UTR, esophageal cancer cells were co-transfected with miR-27a-3p mimics or NC mimics and the TNPO1-wt or TNPO1-mut firefly luciferase reporter vectors using a Lipofectamine 2000 kit (11,668,030, Invitrogen, USA). To assess the binding of E2F7 to QKI promoter, esophageal cancer cells were co-transfected with sh-E2F7 or sh-NC and the QKI-wt or QKI-mut vectors using a Lipofectamine 2000 kit. A renilla luciferase reporter vector was co-transfected to normalize the transfection efficiency. Luciferase activities were detected after transfection for 2 days by a Dual-Luciferase Reporter Assay System (E1980, Promega).

### CCK-8, EdU, and colony formation assays

A Cell Counting Kit-8 (CCK-8, #CK04, Dojindo, Tokyo, Japan) was utilized to assess cell viability. The CCK-8 reagent (10%) was diluted to the working solution and added to a 96-well plate followed by incubation at 37 °C for 2 h. Optical density (OD) values at wavelength of 450 nm were assessed using a microplate reader (#E0226, Beyotime). The proliferation of esophageal cancer cells was measured by EdU and colony formation assays. An EdU Apollo DNA in vitro kit (#C10310, RIBOBIO, Guangzhou, China) was utilized to perform the EDU assay following the manufacturers’ instructions, and then detected under an immunofluorescence microscope. For colony formation assay, EC109 and KYSE150 cells were seeded into six-well plates with 600 cells per well. On the second week after culture, cells were fixed with paraformaldehyde (#P0099, Beyotime) and stained by 0.1% crystal violet (#C0121, Beyotime).

### Wound healing assay

Monolayer esophageal cancer cells were seeded in 6 wells plate, scratched with a sterile 200 μL pipette tip, and then cultured in serum-free medium. Cells were photographed after incubation for 0 and 24 h, and the width of wounds was measured.

### Transwell assays

After transfection, cells (5000 cells/well) were seeded in the inserts pre-equilibrated of 8 μm-pore Transwells (3374, Corning, USA), which were coated with (for invasion) or without Matrigel (#356237, BD Biosciences, USA) (for migration). After incubation for 24 h, cells in the upper chamber were removed by cotton swab while cells on the bottom chamber were fixed in 2% paraformaldehyde for 10 min and stained with crystal violet. Numbers of migrated or invaded cells were counted in six randomly selected fields under a microscope (ECLIPSE Ti, Nikon, Japan).

### Measurement of Fe^2+^, MDA, lipid ROS, and GSH levels

The concentration of Fe^2+^ in esophageal cancer cells was measured using an iron assay kit (ab83366; Abcam) by detecting cell absorbance at 593 nm using a spectrophotometer (Thermo Fisher). The concentration of MDA was detected using a lipid peroxidation MDA assay kit (ab118970; Abcam) by detecting cell absorbance at 532 nm. To detect the level of lipid ROS, cells were stained with 10 μM C11-BODIPY^581/591^ probe (#D3861, Invitrogen) for 30 min. Analysis of C11-BODIPY^581/591^ fluorescence was conducted using a BD Accuri C6 flow cytometer (BD Biosciences). The concentration of GSH was detected using a Glutathione assay kit (CS0260; Sigma) by measuring cell absorbance at 412 nm.

### Transmission electron microscopy

Esophageal cancer cells were fixed with 0.05 M cacodylate buffer (#1313, TIANDZ) containing 2.5% glutaraldehyde (#111–30-8, Merck) and 2% formaldehyde at room temperature for 1 h and then at 4 °C overnight. Next, cells were immersed in 1% osmium tetroxide, rinsed with phosphate buffer, dehydrated with gradient concentrations of ethanol, and embedded in epoxy resin. Next, samples were sliced into 70–80 nm sections using a EM UC7 ultramicrotome (Leica, Wetzlar, Germany) and stained with uranyl acetate and lead citrate. A transmission electron microscope (#H-7650, Hitachi, Tokyo, Japan) at 80 kV was utilized to observe the sections.

### RNA-fluorescence in situ hybridization (FISH)

CircBCAR3 and miR-27a-3p were hybridized with Cy2-labeled probe and Cy5-labeled probe, respectively, according to the manufacturer’s protocols (GenePharma, Shanghai, China). DAPI was used for nuclear staining. A confocal laser scanning microscope (Olympus FV1000) was utilized to observe circBCAR3 and miR-27a-3p in esophageal cancer cells.

### RNA immunoprecipitation (RIP) assay

A Magna RIP RNA-Binding Protein Immunoprecipitation Kit (Millipore, USA) was used following the manufacturer’s instructions. In brief, cells were cross-linked and lysed. Lysate was treated with DNase I for 10 min and centrifuged at 12,000 g for half an hour. Sample was immunoprecipitated with QKI rabbit monoclonal antibody (ab126742, 1:1000, Abcam), or control goat anti-mouse IgG antibody (ab6708, 1:100, Abcam), and added with Protein G magnetic dynabeads (Life Technologies). After washing the beads, the immunoprecipitation was set aside for PCR analysis.

### Xenograft tumor

Nude mice of both sexes (age: 6–8 weeks, weight: 22–25 g) were purchased from HUNAN SJA LABRATORY ANIMAL CO., LTD (Hunan, China). The animal study was approved by the Animal Ethic Review Committees of Ningbo Medical Center Lihuili Hospital, Ningbo University. The EC109 cells stably expressing sh-circBCAR3 or sh-nc were established by infection with corresponding lentivirus vectors (backbone: pGLVU6/Puro; #C06002; GenePharma). 1 × 10^6^ mL^− 1^ (100 μL) cells were subcutaneously inoculated into the nude mice. The tumor volumes had been measured from day 5 to day 25. On day 25, the xenograft tumors were removed surgically, and the tumor weight was detected. Tumor size (width^2^ × length × π/6) was monitored every 5 days. Live imaging was conducted using the Xenogen in vivo imaging system, IVIS-100 (Perkin Elmer, MA, USA). All animal experiments were strictly implemented in compliance with the NIH Guide for the Care and Use of Laboratory Animals.

### Lung metastasis model

BALB/c-nude mice at the age of 6 weeks were inoculated with 100 μL of single-cell suspension of EC109 cells (5 × 10^6^/mL) via tail vein. Forty-five days later, the mice were euthanized. Lung tissues were removed for observation of lung metastasis focus.

### Hematoxylin-eosin staining (H&E)

Tissues were immobilized by 4% paraformaldehyde for 24 h and embedded in paraffin. Five μm sections were collected on microslides. The sections were stained by an H&E staining kit (ab245880, Abcam) following the manufacturer’s instructions.

### Immunohistochemistry (IHC)

The sections from lung tissues were dehydrated and were treated with specific primary antibodies including anti-Vimentin (ab92547; 1:200), anti-E-cadherin (ab40772; 1:500), anti-N-cadherin (ab76011; 1:200), anti-MMP2 (ab86607; 1:100), and anti-MMP9 (ab76003; 1:1000) at 4 °C overnight. Next, 100 μL of HRP conjugated second antibodies IgG (ab6721 and ab6789) at a dilution of 1:2000 were added for 60 min of incubation at 37 °C. The positive staining was visualized by a DAB kit (ab64238, abcam). The staining results were photographed with a microscopy.

### Statistical analysis

Each experiment was technically repeated four times. Data were analyzed with GraphPad Prism 5.0, which was also utilized for drawing the graphs. Difference between two groups was compared using two-tailed, unpaired Student’s t-test. For multiple comparison, one-way or two-way ANOVA was performed. *P* values less than 0.05 indicated statistical significance.

## Results

### Circular RNA circBCAR3 was upregulated in the esophageal cancer tissue and cells

**Fig. 1:**
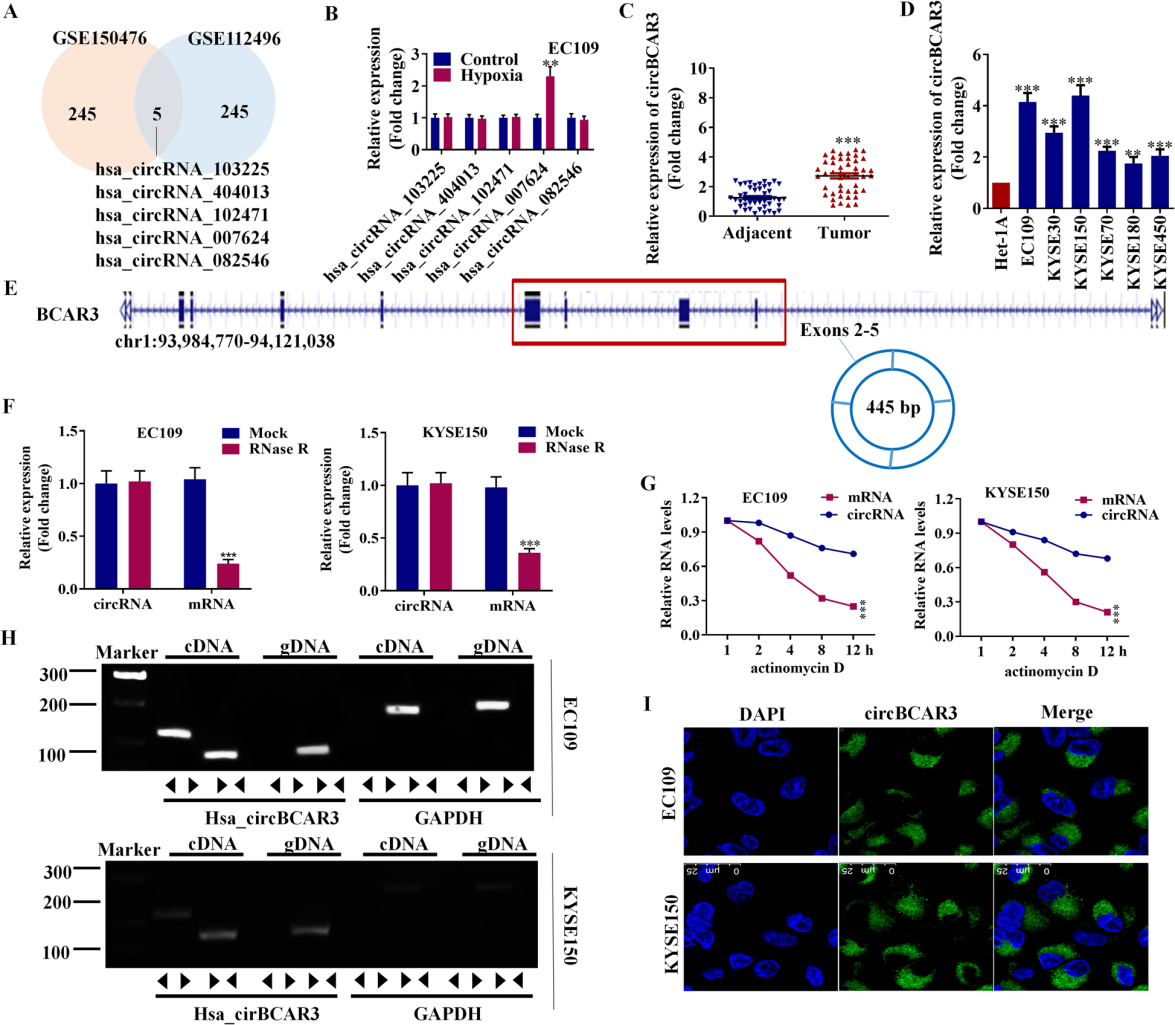
CircBCAR3 exhibits upregulation in esophageal cancer. **A** Differentially expressed circRNAs in esophageal cancer based on both GSE150476 and GSE112496 datasets. **B** Expression of hsa_circRNA_103225, hsa_circRNA_404013, hsa_circRNA_102471, hsa_circRNA_007624, and hsa_circRNA_082546 in hypoxia EC109 cells was revealed by PCR. **C** PCR showed the significant higher expression of circBCAR3 in 45 esophageal carcinoma tissues than adjacent tissues. Student t-test was conducted. **D** PCR showed the significant higher expression of circBCAR3 in esophageal carcinoma cell lines than normal Het-1A cells. One-way ANOVA was conducted. **E** CircBCAR3 is derived from the 2–5 exons of BCAR3 pre-mRNA. **F** PCR analysis of BCAR3 mRNA and circBCAR3 expression in RNase R-treated EC109 and KYSE150 cells. Student t-test was conducted. **G** PCR analysis of BCAR3 mRNA and circBCAR3 expression in actinomycin D-treated EC109 and KYSE150 cells. Two-way ANOVA was conducted. **H** PCR indicated the backsplicing of circBCAR3. CircBCAR3 was amplified by divergent primers in cDNA but not in gDNA with GAPDH as a negative control. **I** FISH revealed the subcellular location and expression of circBCAR3 in EC109 and KYSE150 cells. ** *p* < 0.01, *** *p* < 0.001

To understand the expression of circBCAR3 in esophageal cancer, we first analyzed the expression of circRNAs in esophageal cancer from GEO database. There are five circRNAs that are differentially expressed in esophageal cancer based on both GSE150476 and GSE112496 datasets (Fig. 1A). To explore whether the expression of these dysregulated circRNAs in esophageal cancer can be changed under hypoxia condition, we detected their expression levels in EC109 cells after hypoxia treatment. Hsa_circ_0007624 was found to be upregulated after hypoxia treatment (Fig. 1B). PCR was used to confirm the upregulated expression of hsa_circ_0007624 (circBCAR3) and the results showed that circBCAR3 expression was significantly increased in esophageal tumor samples and cell lines (Fig. 1C, D). As revealed in Table 1, circBCAR3 has close association with tumor grading but has no significant association with other parameters like age, sex, tumor stage, pN category, M status, and family history. The 2, 3, 4, 5 exons of BCAR3 pe-mRNA were back-spliced to form the closed loop construction of circBCAR3 (Fig. 1E). We further detected the stability of circBCAR3 and linear BCAR3 with RNase R. Circular form (circBCAR3) was more resistant to RNase R, while the linear form (BCAR3 mRNA) was significantly decayed (Fig. 1F). In addition, the transcript half-life of circBCAR3 is longer than BCAR3 mRNA after treating with the transcription inhibitor Actinomycin D, which reveals the higher stability of circBCAR3 than BCAR3 mRNA (Fig. 1G). Moreover, to exclude genomic rearrangement of the host gene, convergent primers for BCAR3 mRNA and divergent primers for circBCAR3 were designed. cDNA and genomic DNA were isolated from EC109 and KYSE150 cells and analyzed with agarose gel electrophoresis. CircBCAR3 was amplified by divergent primers in cDNA but not genomic DNA, confirming the presence of circularized BCAR3 exons and excluding trans-splicing products (Fig. 1H). RNA-FISH was subsequently performed in EC109 and KYSE150 cells, and its results revealed that circBCAR3 majorly existed in the cytoplasm of esophageal cancer cells (Fig. 1I).

**Table 1:** Clinicopathological parameters relative to circBCAR3 expression in esophagus cancer tissues

	Evaluable	Low circBCAR3	High circBCAR3	***P*** value
Tumors	45	22	23	
Age group				0.7683
< 65 years	21	11	10	
> 65 years	24	11	13	
Sex				0.6995
Male	38	18	20	
Female	7	4	3	
Tumor stage				0.5420
pT1-pT2	17	7	10	
pT3-pT4	28	15	13	
Grading				**0.0067
G1-G2	25	17	8	
G3-G4	20	5	15	
pN category				> 0.9999
N0-N1	32	16	16	
N2-N3	13	6	7	
M status				> 0.9999
M0	40	20	20	
M1	5	2	3	
Family history				> 0.9999
Yes	4	2	2	
No	41	20	21	

**p < 0.01 indicates significance

### CircBCAR3 knockdown inhibited the proliferation, migration, and invasion of esophageal cancer cells in vitro

**Fig. 2:**
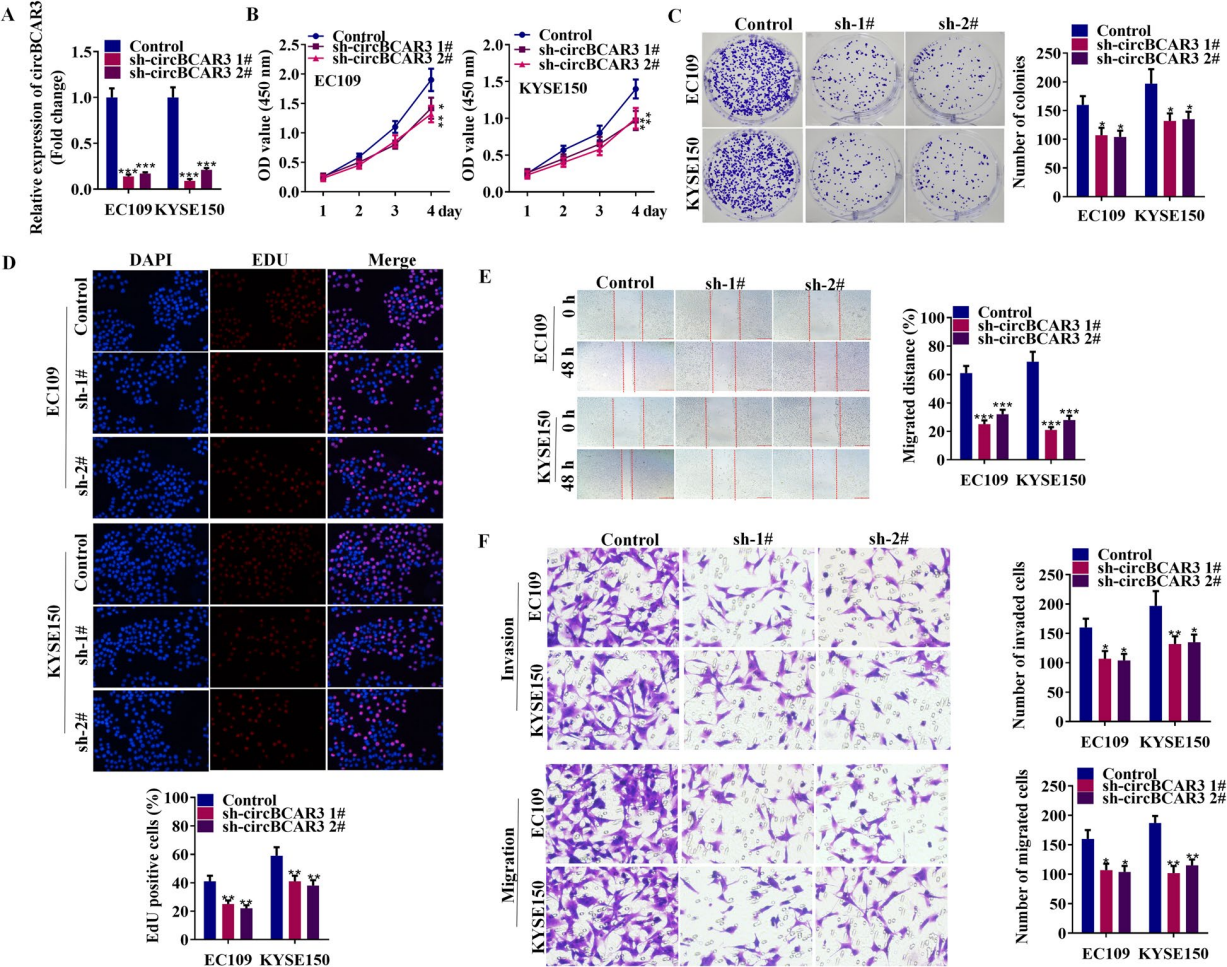
Silenced circBCAR3 inhibits the proliferation and motility of esophageal cancer cells in vitro. **A** PCR analysis of circBCAR3 expression in sh-circBCAR3-treated EC109 and KYSE150 cells. **B** CCK-8, **C** colony formation and **D** EdU assays were utilized to detect the viability and proliferation of EC109 and KYSE150 cells. **E** Wound healing and **F** transwell assays were used to detect the migration and invasion of esophageal cancer cells. * *p* < 0.05, ** *p* < 0.01, *** *p* < 0.001. One-way ANOVA was performed for data analysis in all panels except panel **B**, which is analyzed by two-way ANOVA

To investigate the functions of circBCAR3 in the development of esophageal cancer, we designed two shRNAs to effectively silence circBCAR3 in EC109 and KYSE150 cells (Fig. 2A). The two shRNAs had no significant effects on BCAR3 expression (Supplementary Fig. 1A). CCK-8, colony formation assay, and EdU assay revealed that silenced circBCAR3 repressed the viability and proliferation of EC109 and KYSE150 cells (Fig. 2B-D). Wound healing assay and transwell assays (including migration and invasion) illustrated that the knockdown of circBCAR3 inhibited the migrative and invasive abilities of esophageal cancer cells (Fig. 2E, F). Moreover, knockdown of circBCAR3 suppressed the protein levels of Vimentin, N-cadherin, MMP2, MMP9 and increased the protein level of E-cadherin (Supplementary Fig. 1B).

### CircBCAR3 knockdown inhibited the ferroptosis of esophageal cancer cells

**Fig. 3:**
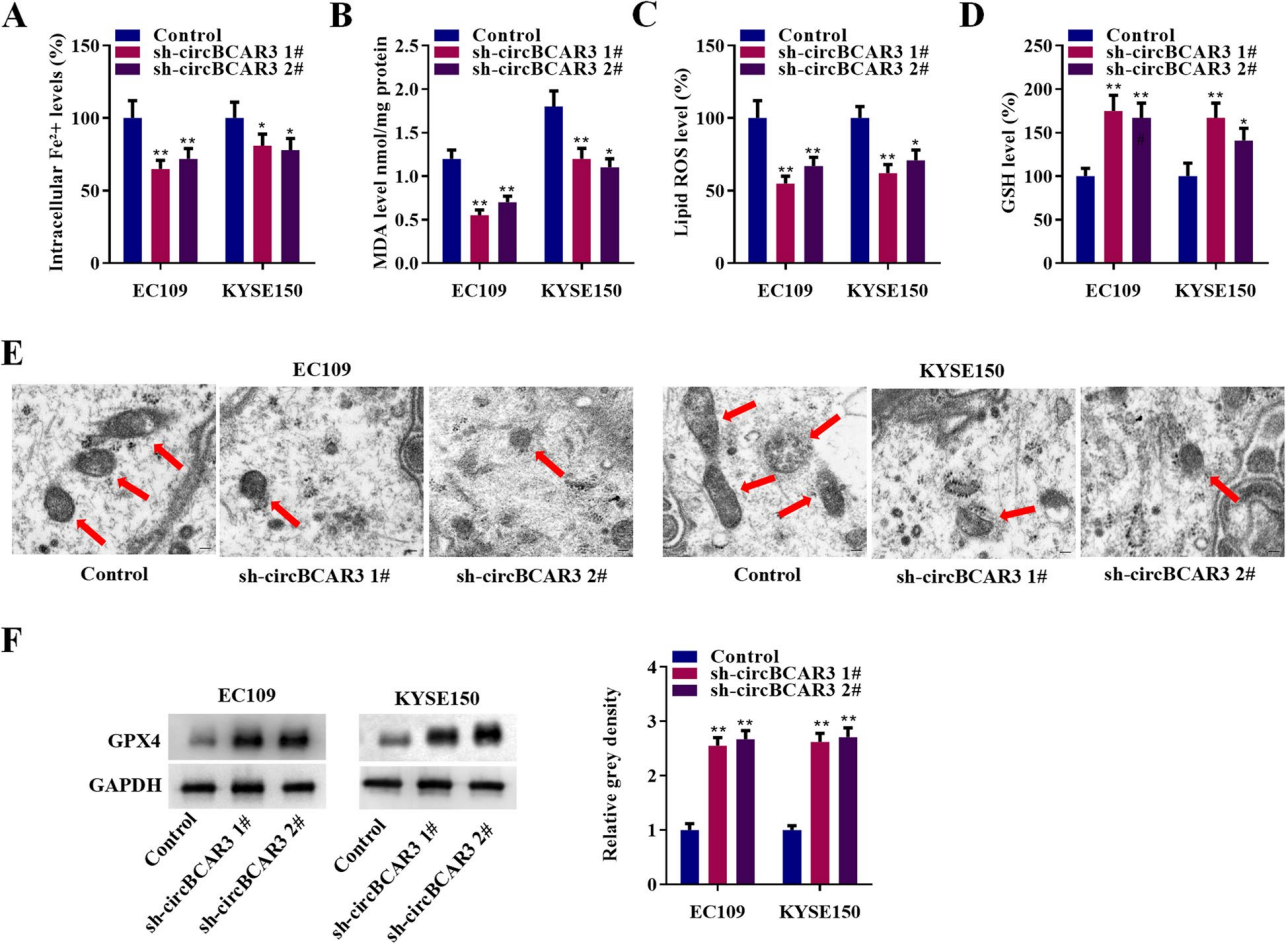
Silenced circBCAR3 inhibits the ferroptosis of esophageal cancer cells in vitro. **A** Intracellular Fe^2+^ levels in EC109 and KYSE150 cell after transfection with circBCAR3 shRNA were detected with an iron assay kit. **B** MDA level was detected with a lipid peroxidation MDA assay kit. **C** Lipid ROS levels were assessed using C11-BODIPY^581/591^ probe. Fluorescence analysis was conducted using flow cytometry. **D** GSH levels were measured with a Glutathione assay kit. **E** Transmission electron microscopy was utilized to observe morphological changes in EC109 and KYSE150 cell after transfection with circBCAR3 shRNA. **F** Western blotting was performed to examine GPX4 protein. * *p* < 0.05, ** *p* < 0.01. One-way ANOVA was performed

Effects of circBCAR3 knockdown on ferroptosis were further explored. As revealed in Fig. 3A-D, sh-circBCAR3 reduced intracellular Fe^2+^, MDA, lipid ROS, and increased GSH levels. The typical morphological changes of reduced ferroptosis in esophageal cancer cells by sh-circBCAR3 was observed by transmission electron microscopy (Fig. 3E). GPX4 protein levels were increased by silencing of circBCAR3, as revealed by western blotting in Fig. 3F.

### CircBCAR3 knockdown reversed the effect of hypoxia on the proliferation, migration, invasion, and ferroptosis of esophageal cancer cells

**Fig. 4:**
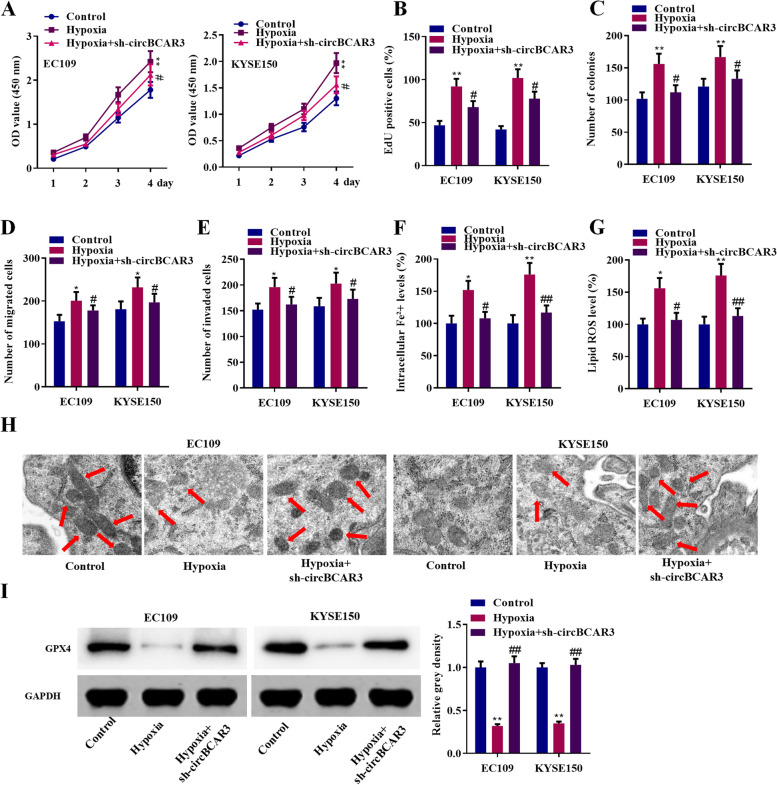
Knockdown of circBCAR3 reversed the effects of hypoxia on esophageal cancer cells in vitro. **A** CCK-8, **B** colony formation and **C** EdU assays were performed to reveal the viability and proliferation of EC109 and KYSE150 cells after hypoxia treatment or cotreatment of hypoxia+sh-circBCAR3. **D** Wound healing and **E** transwell assays were used to assess the migration and invasion of esophageal cancer Cells. **F** Fe^2+^ levels and **G** lipid ROS levels were assessed. **H** Cell morphological changes were observed using transmission electron microscopy to confirm ferroptosis. **I** GPX4 protein was assessed by western blotting. * *p* < 0.05, ** *p* < 0.01 vs control, ^#^*p* < 0.05, ^##^*p* < 0.01 vs hypoxia. Data for CCK-8 assay were analyzed using two-way ANOVA and data for the remaining assays were analyzed using one-way ANOVA

Function studies were again performed to evaluate whether circBCAR3 mediates the hypoxia induced alteration in malignant behaviors of esophageal cancer cells. The results indicated that hypoxia treatment significantly promoted the proliferation, migration, invasion, and ferroptosis of esophageal cancer cells. CircBCAR3 knockdown notably reversed these effects of hypoxia (Fig. 4).

### Silenced circBCAR3 inhibited the tumor growth and metastasis of esophageal cancer in vivo

**Fig. 5:**
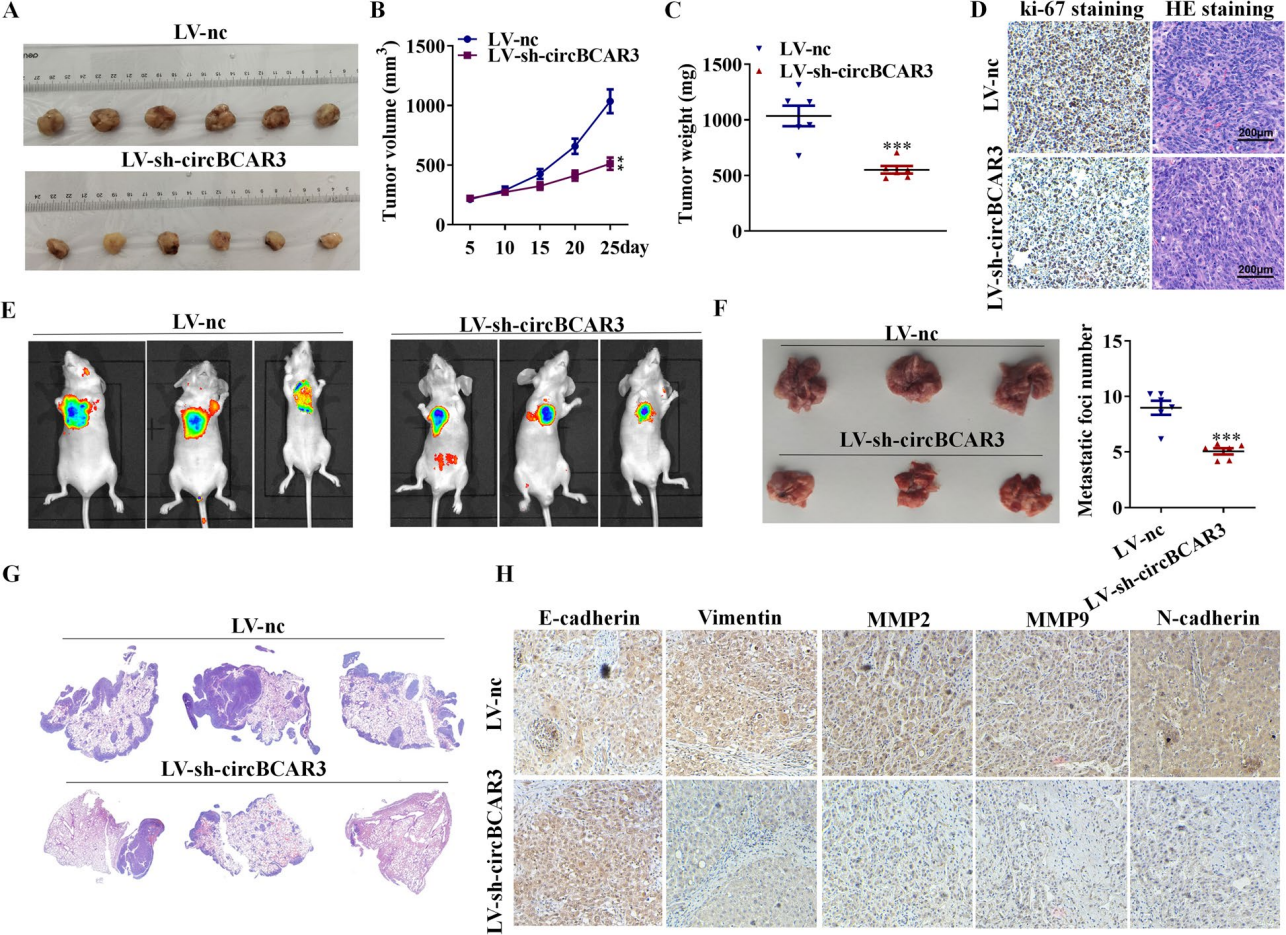
Knockdown of circBCAR3 inhibits the esophageal cancer growth and metastasis in vivo. **A** Photographs of xenograft tumors formed by EC109 cells stably expressing sh-circBCAR3 and sh-NC. **B** The tumor volume of mice in the sh-circBCAR3 and the control group was shown. **C** The tumor weight was shown. **D** Ki-67 immunohistochemistry staining and H&E staining of xenograft tumors. **E** Xenogen images illustrate the growth of the tumor inside the mice. **F** The lung tissues with metastasis from mice were shown. **G** H&E staining was performed to reveal the metastatic foci in lung tissues. **H** IHC was used to evaluate the expression of EMT markers including Vimentin, N-cadherin, MMP-2, MMP-9, and E-cadherin in the lung tissues. *n* = 6. ** *p* < 0.01. Two-way ANOVA was performed for difference comparison in **B**, while student’s t test was used for **C** and **F**

To study the role of circBCAR3 in tumor growth and metastasis in vivo, we established esophageal xenograft in mice by injection of cells stably expressing sh-nc and sh-circBCAR3. Supplementary Fig. 1C revealed that circBCAR3 expression was reduced by infection of lv-sh- circBCAR3 in EC109 cells. The results demonstrated that circBCAR3 deficiency decreased the volume and weight of esophageal tumor in mice (Fig. 5A-C). Moreover, the results of IHC staining demonstrated that silenced circBCAR3 reduced the expression of proliferative marker ki67. H&E staining results indicated that cells distributed densely, and cell morphology was normal in tumors of the sh-nc group, while the number of tumor cells decreased and there were more shrank nuclei in tumors of the sh-circBCAR3 group (Fig. 5D). Thereafter, we established a tumor metastasis model to monitor lung metastasis via tail vein injection of esophageal cancer cells. The bioluminescence image showed that silenced circBCAR3 induced tumor inhibition in mice, as evidenced by the decreased bioluminescent intensity (Fig. 5E). Silenced circBCAR3 suppressed the lung metastasis of esophageal cancer compared with those in the control group (Fig. 5F). H&E staining results further indicated this trend (Fig. 5G). The IHC staining suggested that knockdown of circBCAR3 inhibited the expression of vimentin, N-cadherin, MMP-2, MMP-9 and promoted that of E-cadherin in lung tissues (Fig. 5H). These data suggested that circBCAR3 knockdown attenuates the esophageal tumor growth and metastasis in vivo.

### CircBCAR3 negatively regulates miR-27a-3p in esophageal cancer cells

**Fig. 6:**
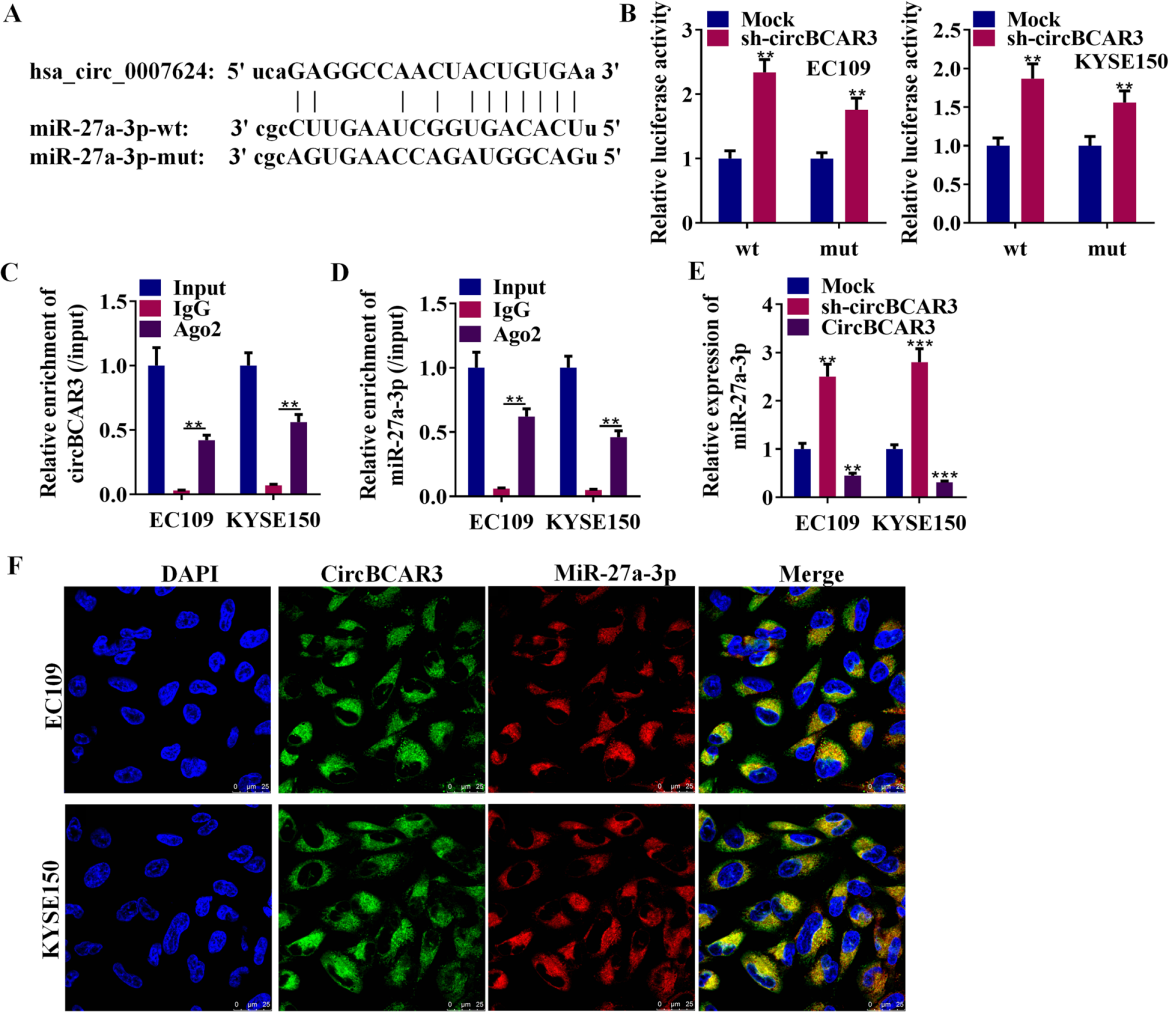
CircBCAR3 acts as the sponge of miR-27a-3p in esophageal cancer cells. **A** Binding sequences of miR-27a-3p on circBCAR3. The complementary bases are linked in vertical line. **B** Luciferase reporter assay was performed for revealing the binding between miR-27a-3p and circBCAR3. **C**, **D** RIP assay was conducted for detecting the binding of circBCAR3 and miR-27a-3p to the Ago antibody. **E** PCR tested miR-27a-3p expression in EC109 and KYSE150 cells with circBCAR3 knockdown or overexpression. **F** FISH assay revealed the expression and subcellular location of miR-27a-3p and circBCAR3 in EC109 and KYSE150 cells. ** *p* < 0.01, *** *p* < 0.001

Potential miRNAs binding to circBCAR3 were predicted from the ENCORI database. We focused on miR-27a-3p because of its high ranking. Binding site of miR-27a-3p and circBCAR3 was shown in Fig. 6A. Sequences of miR-27a-3p were mutated for the following luciferase reporter assay. The luciferase assay results showed that circBCAR3 negatively regulates wild type miR-27a-3p and had no binding relationship with mutant miR-27a-3p (Fig. 6B). RIP assay revealed that Ago2, an effector of miRNA mechanism, can bind with miR-27a-3p and circBCAR3 (Fig. 6C, D). After knockdown of circBCAR3, enrichment of miR-27a-3p in Ago2-formed complexes was decreased (Supplementary Fig. 1D). MiR-27a-3p was downregulated in esophageal cancer tissues (Supplementary Fig. 1E) and its expression was decreased by hypoxia (Supplementary Fig. 1F). Transfection of circBCAR3 shRNAs into EC109 and KYSE150 cells increased the miR-27a-3p expression level, while overexpressed circBCAR3 in EC109 and KYSE150 cells decreased the miR-27a-3p expression level, compared with the control groups (Fig. 6E). In addition, to identify the subcellular distribution of circBCAR3 and miR-27a-3p in esophageal cancer cells, RNA-FISH assay was performed and indicated that circBCAR3 and miR-27a-3p were both located in the cytoplasm (Fig. 6F).

### TNPO1 functions as the target of miR-27a-3p in esophageal cancer cells

**Fig. 7:**
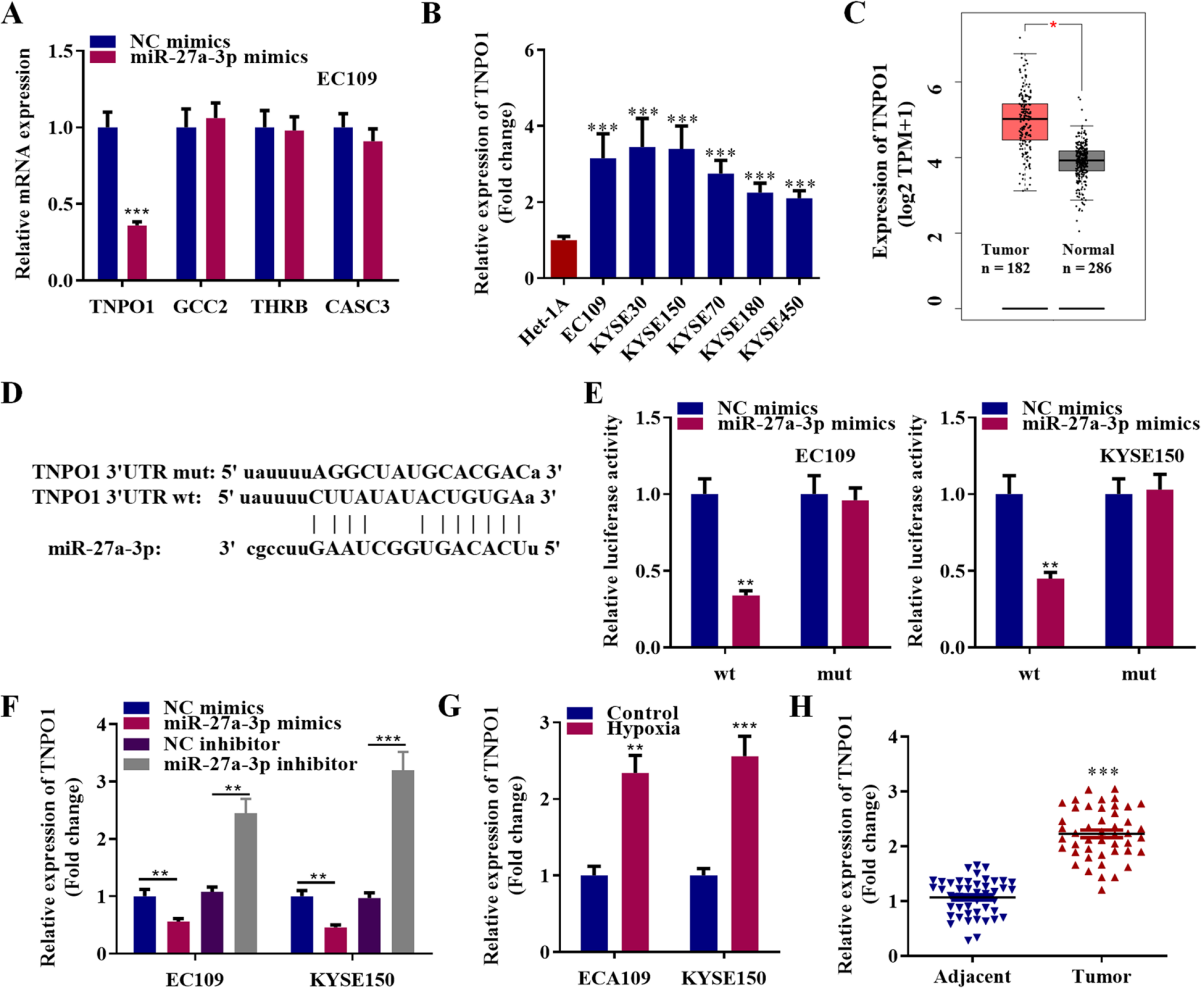
TNPO1 functions as the target of miR-27a-3p in esophageal cancer cells. **A** Expression of TNPO1, GCC2, THRB, CASC3 in EC109 cells by transfection with miR-27a-3p mimics was assessed by PCR analysis. **B** PCR analysis of expression of TNPO1 in six esophageal cancer cells and control Het-1A cell line. **C** The expression of TNPO1 in 182 esophageal cancer tissues was obtained from the GEPIA database. **D** Binding site of miR-27a-3p on TNPO1 was obtained from ENCORI database. Complementary bases are linked in vertical line. **E** Regulation of miR-27a-3p on TNPO1 3’UTR was confirmed with luciferase reporter assays. **F** PCR revealed TNPO1 expression in EC109 and KYSE150 cells by treatment of miR-27a-3p mimics or inhibitor. **G** Expression of TNPO1 in hypoxic EC109 and KYSE150 cells was revealed by PCR analysis. **H** PCR showed the significant high expression of TNPO1 in 45 esophageal carcinoma tissues. * *p* < 0.05, ** *p* < 0.01, *** *p* < 0.001. Student’s t test was performed

Based on ENCORI database, TNPO1, GCC2, THRB, and CASC3 were identified as the potential targets of miR-27a-3p. Expression of the four targets under the transfection of miR-27a-3p mimics was detected. The results showed that miR-27a-3p caused the decrease of TNPO1 expression in EC109 cells (Fig. 7A). TNPO1 is significantly upregulated in six esophageal cancer cells compared with control Het-1A cell line (Fig. 7B). We also found that the expression of TNPO1 was higher in 182 esophageal cancer tissues than that in 286 adjacent non-tumor tissues from GEPIA database (Fig. 7C). TNPO1 3’UTR was complementary to the sequences of miR-27a-3p, as predicted by ENCORI database (Fig. 7D). TNPO1 3’UTR wild type and mutant luciferase reporters were constructed for luciferase assays, which confirmed that TNPO1 was a direct target of miR-27a-3p (Fig. 7E). MiR-27a-3p mimics effectively reduced TNPO1 expression level, while the inhibitor of miR-27a-3p significantly enhanced the TNPO1 expression level (Fig. 7F). CircBCAR3 silencing reduced TNPO1 expression while pcDNA-circBCAR3 increased TNPO1 expression (Supplementary Fig. 1G). Hypoxia can induce the upregulation of TNPO1 in esophageal cancer cells (Fig. 7G). PCR results showed TNPO1 was significantly high expressed in 45 esophageal carcinoma tissues compared to that in 45 adjacent non-tumor tissues (Fig. 7H).

### TNPO1 overexpression rescued the silenced circBCAR3-mediated inhibition of proliferation, migration, invasion, and ferroptosis of esophageal cancer cells in vitro

**Fig. 8:**
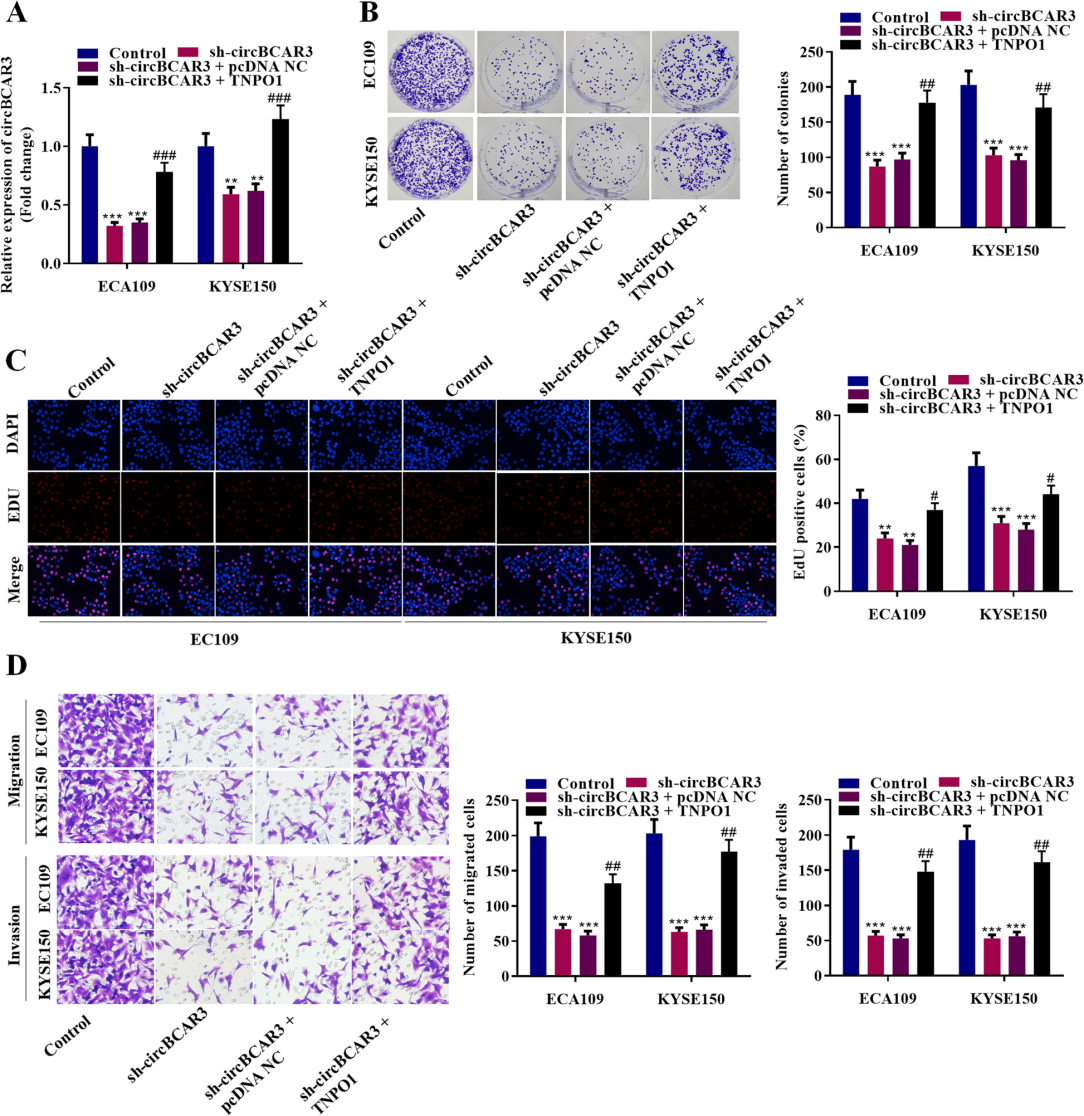
TNPO1 reversed the circBCAR3 silencing-induced inhibition of proliferation and motility of esophageal cancer cells. **A** PCR analysis of TNPO1 expression in EC109 and KYSE150 cells after the cotransfection of circBCAR3 shRNA+pcDNA-TNPO1. **B** EdU, **C** colony formation, and **D** Transwell migration and invasion assays were performed in EC109 and KYSE150 after the cotransfection of circBCAR3 shRNA+pcDNA-TNPO1 to measure cell proliferation, migration, and invasion. ** *p* < 0.01, *** *p* < 0.001, ^#^*p* < 0.01, ^##^*p* < 0.01, ^###^*p* < 0.001. One-way ANOVA was performed

**Fig. 9:**
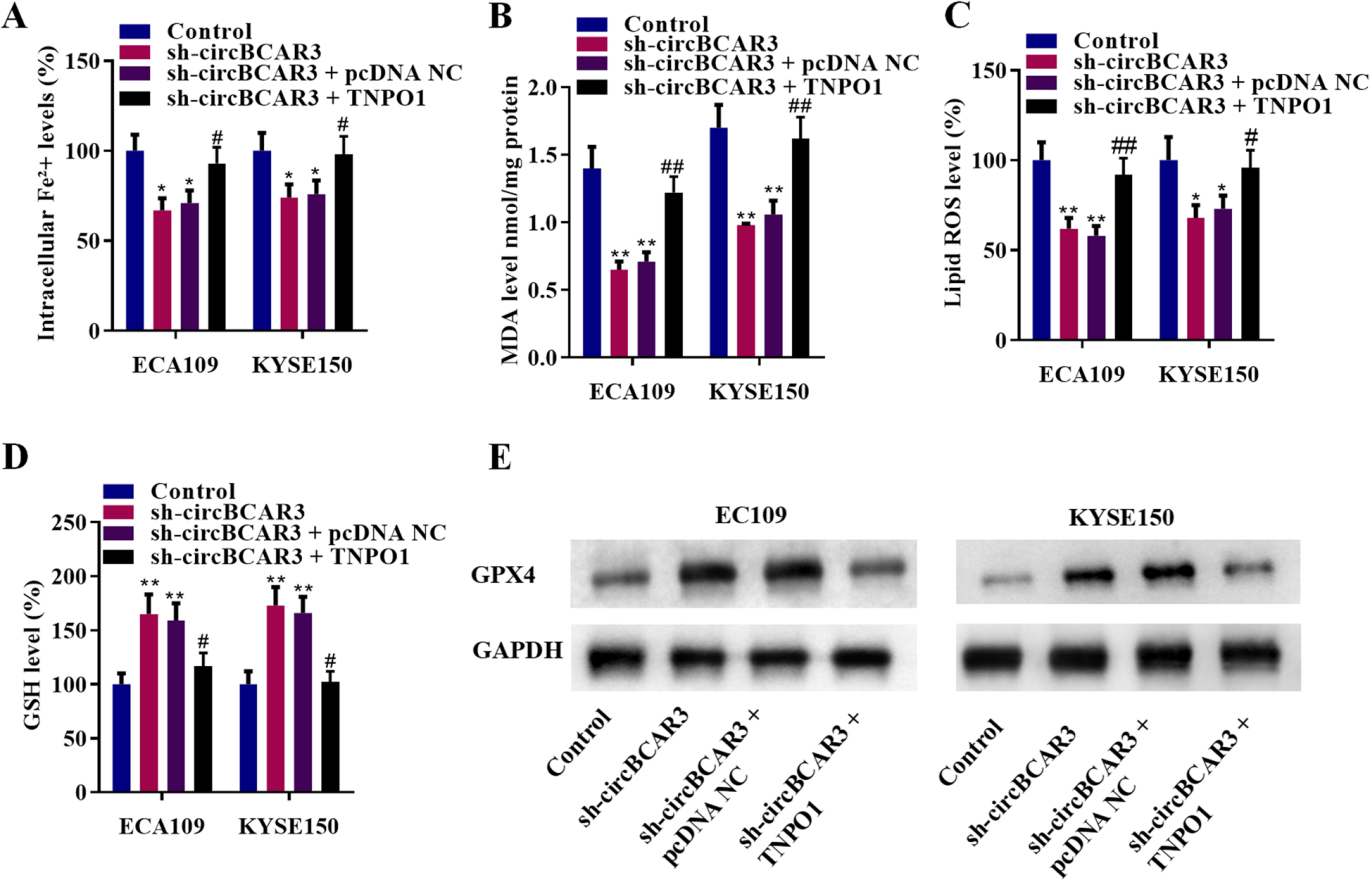
TNPO1 rescued the sh-circBCAR3-induced ferroptosis inhibition in esophageal cancer cells. **A** Intracellular Fe^2+^ levels in EC109 and KYSE150 cells after cotransfection with sh-circBCAR3 + pcDNA-TNPO1 were detected with an iron assay kit. **B** MDA level was detected with a lipid peroxidation MDA assay kit. **C** Lipid ROS levels were assessed using C11-BODIPY^581/591^ probe. Fluorescence analysis was conducted using flow cytometry. **D** GSH levels were measured with a Glutathione assay kit. **E** Western blotting of GPX4 protein. * *p* < 0.05, ** *p* < 0.01, ^#^*p* < 0.01, ^##^*p* < 0.01. One-way ANOVA was performed

We designed the overexpression vector of TNPO1 and found that it effectively rescued the suppressive effects of sh-circBCAR3 on TNPO1 expression (Fig. 8A). Moreover, EdU assay and colony formation assay showed that cell viability and proliferation were decreased by circBCAR3 shRNA and further increased by TNPO1 overexpression (Fig. 8B-C). Transwell migration and invasion assays illustrated that TNPO1 overexpression rescued the suppressive effects of knockdown of circBCAR3 on cell migrative and invasive abilities (Fig. 8D). Figure 9 revealed that the inhibitory effects of sh-circBCAR3 on ferroptosis of esophageal cancer cells were rescued by TNPO1 overexpression, as evidenced by the pcDNA-TNPO1-induced increase of intracellular Fe^2+^, MDA, lipid ROS, and decrease of GSH and GPX4 levels.

### Splicing factor QKI accelerates the biogenesis of circBCAR3

**Fig. 10:**
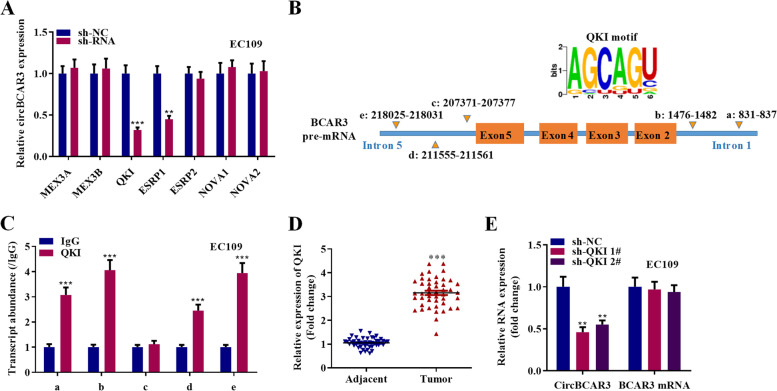
Splicing factor QKI accelerates the biogenesis of circBCAR3. **A** PCR analysis of circBCAR3 expression in EC109 cells after transfection with shRNAs against various splicing factors. **B** Multiple QKI binding sequences were found in the flanking of circBCAR3. **C** RIP assay using QKI antibody was conducted for assessing the binding of circBCAR3 and QKI. **D** PCR examined the expression of QKI in 45 paired esophageal cancer and adjacent nontumor tissues. **E** Effects of silenced QKI on expression of circBCAR3 and linear BCAR3 were revealed by PCR analysis. ** *p* < 0.01, *** *p* < 0.001

To identify the regulatory mechanism of circBCAR3 formation, we designed shRNAs against splicing factors including MEX3A, MEX3B, QKI, ESRP1, ESRP2, NOVA1, NOVA2 and detected circBCAR3 expression after transfections with these shRNAs. Results of PCR analysis illustrated that splicing factors QKI and ESRP1 negatively regulated circBCAR3 expression in esophageal cancer cells while other splicing factors had no significant effects on circBCAR3 expression (Fig. 10A). We next revealed five QKI binding sequences flanking the circBCAR3-forming exons of BCAR3 (Fig. 10B). RIP assay using QKI antibody demonstrated that QKI bound with BCAR3 pre-mRNA at sites a, b, e, and f (Fig. 10C). Furthermore, QKI is upregulated in our collected 45 esophageal cancer tissues (Fig. 10D). QKI knockdown suppressed circBCAR3 expression and had no effects on BCAR3 mRNA expression (Fig. 10E). Moreover, sh-QKI increased miR-27a-3p expression and decreased TNPO1 expression (Supplementary Fig. 1H). These findings suggested that splicing factor QKI accelerates the biogenesis of circBCAR3 via binding sites in introns.

### Hypoxia induced expression of E2F7 activated the transcription of QKI

**Fig. 11:**
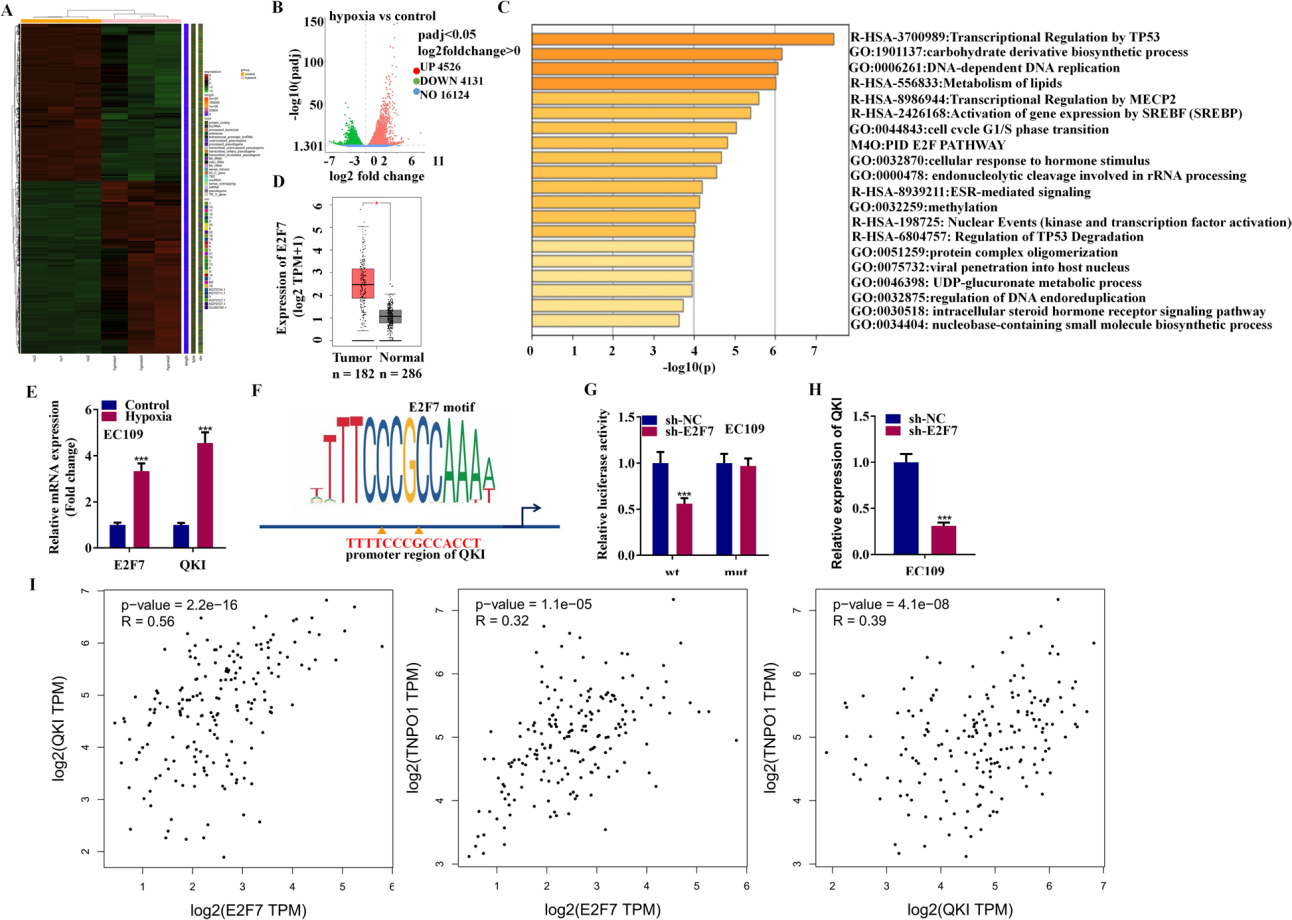
Hypoxia-induced E2F7 activated the transcription of QKI. **A** The heatmap and **B** volcano plot showing the dysregulated expression of genes in esophageal cancer cells under hypoxia treatment. **C** Metascape was used to perform the GO enrichment analysis. **D** The expression of E2F7 in 182 esophageal cancer tissues was obtained from the GEPIA database. **E** PCR analysis of E2F7 and QKI expression in EC109 cells under hypoxia. **F** The DNA motif of E2F7 and its potential binding site in the promotor of QKI were predicted from JASPAR website. **G** Luciferase assay was performed to detect whether E2F7 silencing can change the activity of the reporter vector carrying wild or mutant fragments of QKI promoter. **H** PCR analysis of QKI expression after E2F7 knockdown. **I** GEPIA database was used to evaluate the expression correlation between E2F7 and QKI, E2F7 and TNPO1, QKI and TNPO1 in 182 esophageal cancer tissues. *** *p* < 0.001

We have confirmed that circBCAR3 is highly expressed in esophageal cancer and is upregulated by hypoxia treatment. How hypoxia induces the elevated expression of circBCAR3 remains unclear. Thus, we performed the RNA-seq to screen the dysregulated genes, as shown in the heat map and Volcano Plot (Fig. 11A, B). The following enrichment analysis revealed that genes from E2F family were enriched (Fig. 11C). We searched the GEPIA database to explore the expression of those E2Fs in esophageal cancer. Interestingly, we found that E2F7 was notably highly expressed in esophageal cancer (Fig. 11D). PCR analysis confirmed that both E2F7 and QKI were upregulated in EC109 cells after hypoxia treatment (Fig. 11E). As E2F7 is a transcription activator, we assume that E2F7 may regulate the transcription of QKI. In the promoter of QKI, we found a potential binding site of E2F7 based on JASPR online database (Fig. 11F). Thereafter, we subcloned the wild and mutant forms of this binding site into the pGL3 vector. Luciferase assay was carried out. The results indicated that E2F7 promoted the luciferase activity of the reporter vector carrying the wild binding site but not the mutant one (Fig. 11G). Furthermore, E2F7 knockdown reduced QKI mRNA expression level (Fig. 11H). E2F7 decreased circBCAR3, TNPO1 expression and increased miR-27a-3p expression (Supplementary Fig. 1I). We searched the GEPIA database to check the expression correlation between these key molecules in 182 esophageal cancer tissues and the results revealed a positive expression correlation between E2F7 and QKI, E2F7 and TNPO1, QKI and TNPO1 (Fig. 11I).

## Discussion

CircRNAs are regulated by hypoxia in cancers [37]. The present study identified that circBCAR3 is upregulated in esophageal cancer and its expression can be further increased by hypoxia. Hypoxia induces the epithelial mesenchymal transition process, which is responsible for cell migration and invasion in oral cancer [38] and breast cancer [39]. Similarly, we found that hypoxia causes the increase of proliferative, migrative, and invasive abilities of esophageal cancer cells. Excess iron has close association with carcinogenesis [40]. Cancers are considered to originate as side-effects of exposure to iron [41] and oxygen [42] for a long time. Hypoxia/regeneration can enhance ferrptosis in vitro [43]. In the present study, ferrptosis is promoted by hypoxia in esophageal cancer cells. CircBCAR3 knockdown exerts negative effects on the proliferation, migration, invasion, and ferrptosis of esophageal cancer cells. The hypoxia-stimulated effects on esophageal cancer cells can be partially abrogated by circBCAR3. Results of in vivo studies showed that circBCAR3 suppressed esophageal xenograft growth and metastasis in mice.

Alternative splicing of pre-mRNA is an important biological feature in eukaryotes [44]. QKI has been indicated to regulate pre-mRNA splicing [45]. Of note, insertion of QKI motifs can induce circRNA formation. Secondary structure within pre-mRNAs brings forming exons of circRNAs into close proximity and consequently promotes circRNA biogenesis [46]. A previous study indicated that QKI targets the introns flanking the circRNA-forming exons of SMARCA5 [47] or NDUFB2 [48] to promote circRNA formation. In accordance with these studies, we found that QKI has binding sites with the introns 1 and 5 of BCAR3 pre-mRNA and enhanced circBCAR3 biogenesis by binding to recognition elements within introns in the vicinity of circBCAR3-forming splice sites. It can be inferred that QKI promotes circBCAR3 biogenesis by bringing the 1, 5 exons into close proximity. Although our study lacks investigations on the functions of QKI in esophageal cancer, it has been indicated to be positively associated with tumor metastasis and prognosis in patients with this cancer and to promote esophageal cancer cell proliferation in vitro [49]. We also found that hypoxia can increase QKI expression in esophageal cancer cells, which is in consistent with a previous study demonstrating the hypoxia-induced increase of QKI in rats [50]. Furthermore, KEGG enrich analysis revealed the involvement of the E2F pathway in hypoxic esophageal cancer cells. Interestingly, E2F7 is upregulated in esophageal cancer, as evidenced by GEPIA database and a previous study [51]. E2F7 is a known transcriptional factor to activate [52] or suppress [53] downstream genes. Our findings demonstrated that hypoxia-induced E2F7 promoted the upregulation of QKI at the transcriptional level, which further contributed to circBCAR3 biogenesis. Hypoxia induces the upregulation of HIF proteins [54]. HIF-2α is involved in the transcriptional activity of E2F [55]. Based on hTFtarget database [56], HIF-1α is a transcriptional factor for E2F7, which may explain the hypoxia-induced E2F7 in esophagus cancer cells.

The circRNA-miRNA-mRNA pattern under hypoxia condition is involved in cancer progression, for instance, circDENND4C under hypoxia promotes glycolysis, migration, and invasion of breast cancer cells via interacting with miR-200b and miR-200c [57]. The hypoxia-induced circ-0000977 modulates the immune escape of prostate cancer cells via the miR-153/HIF1A/ADAM10 axis [58]. We found that circBCAR3 interacted with miR-27a-3p by the ceRNA mechanism to upregulate TNPO1 in esophageal cancer cells. MiR-27a-3p expression is increased in esophageal cancer cells with knockdown of TP53 [59], which regulates ferroptosis sensitivity [60]. Our experimental results revealed that TNPO1 was upregulated in esophageal cancer tissues and showed positive expression correlation with QKI and E2F7. Similarly, Pauline J van der Watt et al. also revealed the increased TNPO1 expression in this cancer and indicated it as a biomarker that has high diagnostic capacity with an area under the curve of 0.963 and sensitivity of 95.3% sensitivity at 87.5% specificity [61]. At the cellular levels, TNPO1 reversed the inhibitory impacts of circBCAR3 knockdown on the proliferation, migration, invasion, and ferroptosis of esophageal cancer cells. These findings indicated the potential application of molecular therapy targeting TNPO1 in esophageal cancer.

However, the detailed mechanism underlying TNPO1 in esophageal cancer is limited. TNPO1 has been identified as a binding partner of carbonic anhydrase IX, a transmembrane protein affecting cell survival in hypoxic tumors [62]. Our further study will focus on the potential interaction of TNPO1 and carbonic anhydrase IX in hypoxic esophageal cancer. Moreover, hypoxic microenvironment gives rise to the EMT process in cancers [63, 64]. Considering the substantial regulation of circRNA biogenesis during EMT [30] and the E-cadherin-mediated ferroptosis suppression [65], whether EMT is activated in hypoxic esophageal cancer and whether its activation has association with ferroptosis will be explored in our further research.

## Conclusion

We innovatively demonstrated the oncogenic role of circBCAR3 in esophageal cancer by promoting cancer cell proliferation, migration, invasion, and ferroptosis and by promoting esophageal tumorigenesis and metastasis in mice. At the molecular level, hypoxia-induced E2F7 transcriptionally activates splicing factor QKI, which promotes circBCAR3 biogenesis by binding to intronic QKI response elements flanking circBCAR3-forming exons. CircBCAR3 interacts with miR-27a-3p to upregulate TNPO1, thus exerting its biological functions in esophageal cancer cells. These data suggest circBCAR3 as a potential marker in research and treatment of esophageal cancer.

## Supplementary Information

**Additional file 1: Supplementary Figure 1.** (A) Expression of circBCAR3 in EC109 and KYSE150 cells after transfecting sh-circBCAR3 1/2# was assessed by PCR. (B) Protein levels of E-cadherin, Vimentin, N-cadherin, MMP2, MMP9, and GAPDH in EC109 and KYSE150 cells after transfecting sh-circBCAR3 1/2# was assessed by PCR. (C) Relative expression of circBCAR3 in EC109 cells after infection with lv-sh-circBCAR3 was assessed by PCR. (D) Relative enrichment of miR-27a-3p in Ago2-formed complexes in EC109 and KYSE150 cells after knockdown of sh-circBCAR3 1/2# was assessed by RIP assay. (E) MiR-27a-3p expression in esophageal cancer tissues was normalized to that in adjacent tissues. (F) MiR-27a-3p expression in EC109 cells by hypoxia was assessed by PCR. (G) TNPO1 expression in EC109 and KYSE150 cells after transfection with sh-circBCAR3 and pcDNA3.1-circBCAR3 was assessed by PCR. (H) Relative expression of miR-27a-3p and TNPO1 in EC109 cells after transfection with sh-QKI 1/2#. (I) Relative expression of circBCAR3, miR-27a-3p, and TNPO1 in EC109 cells after transfection with sh-E2F7 1/2#. * *p* < 0.05, ** *p* < 0.01, *** *p* < 0.001.

## Data Availability

All materials underlying this study are available from the corresponding author on the basis of a material transfer agreement.

## Abbreviations

BCAR3: Breast cancer antiestrogen resistance 3
CCK-8: Cell counting kit-8
circRNA: Circular RNA
E2F7: E2F transcription factor 7
EdU: 5-Ethynyl-2′- deoxyuridine
ENCORI: The encyclopedia of RNA interactomes
FISH: Fluorescence in situ hybridization
GEO: Gene expression omnibus
GEPIA: Gene expression profiling interactive analysis
GO: Gene oncology
GPX4: Glutathione peroxidase 4
GSH: Glutathione
H&E: Hematoxylin-eosin staining
HRP: Horseradish peroxidase
IHC: Immunohistochemistry
KEGG: Kyoto encyclopedia of genes and genomes
MDA: Malonaldehyde
miRNA: MicroRNA
ncRNA: Noncoding RNA
NPO1: Transportin-1
OD: Optical density
PVDF: Polyvinylidene difluoride
QKI: Splicing factor quaking
qRT-PCR: Quantitative real-time polymerase chain reaction
RBP: RNA binding protein
ROS: Reactive oxygen species
SDS-PAGE: Sodium dodecyl sulfate-polyacrylamide gel electrophoresis
TRIP: RNA immunoprecipitation
UTR: Untranslated region
