# Supplementary figures and images for: CircBCAR3 accelerates esophageal cancer tumorigenesis and metastasis via sponging miR-27a-3p
Source: Mol Cancer. 2022 Jul 15;21:145. doi: 10.1186/s12943-022-01615-8 (PMC9284725; doi:10.1186/s12943-022-01615-8)

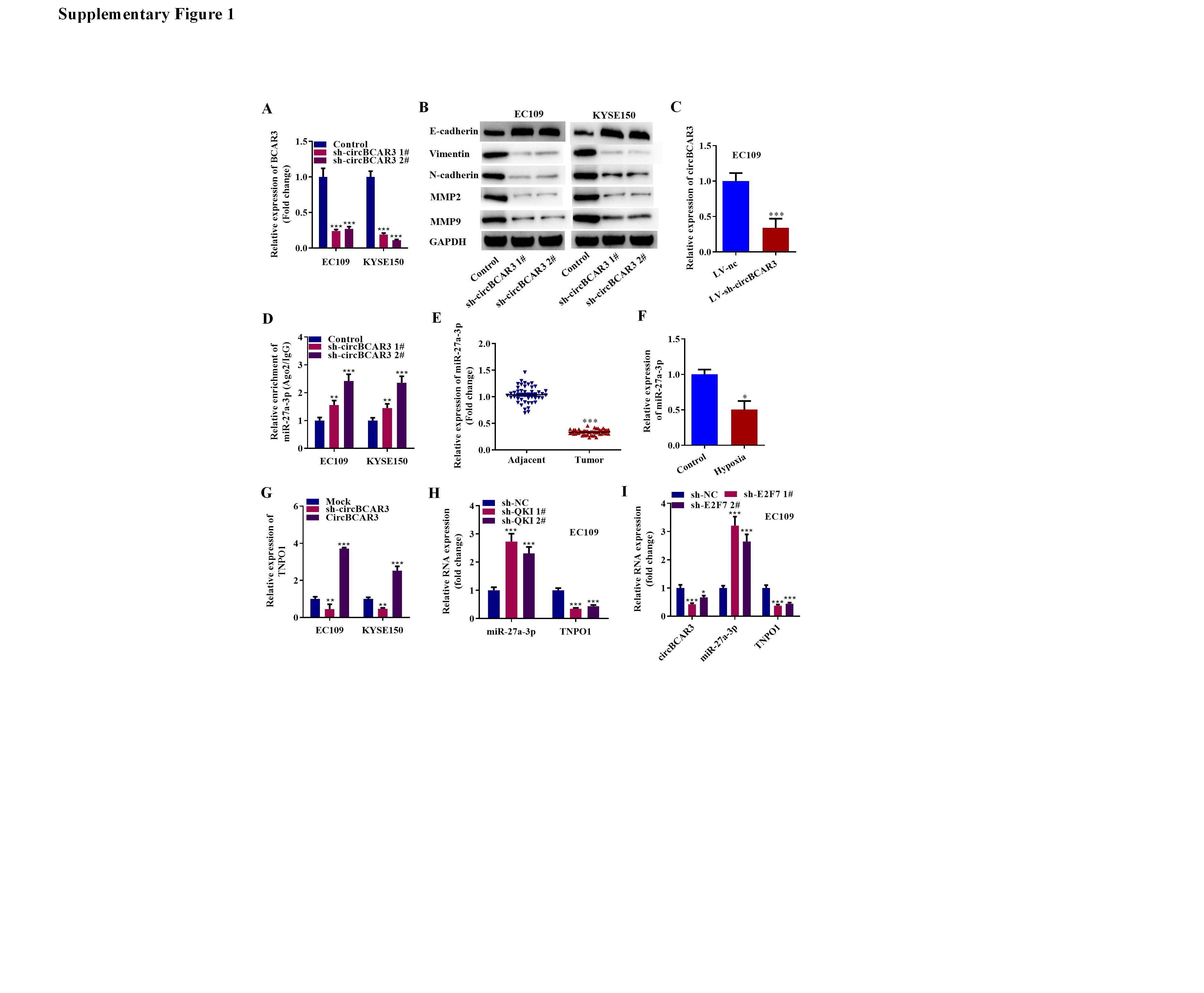

Supplement: Supplementary file 1 — Additional file 1: Supplementary Figure 1. (A) Expression of circBCAR3 in EC109 and KYSE150 cells after transfecting sh-circBCAR3 1/2# was assessed by PCR. (B) Protein levels of E-cadherin, Vimentin, N-cadherin, MMP2, MMP9, and GAPDH in EC109 and KYSE150 cells after transfecting sh-circBCAR3 1/2# was assessed by PCR. (C) Relative expression of circBCAR3 in EC109 cells after infection with lv-sh-circBCAR3 was assessed by PCR. (D) Relative enrichment of miR-27a-3p in Ago2-formed complexes in EC109 and KYSE150 cells after knockdown of sh-circBCAR3 1/2# was assessed by RIP assay. (E) MiR-27a-3p expression in esophageal cancer tissues was normalized to that in adjacent tissues. (F) MiR-27a-3p expression in EC109 cells by hypoxia was assessed by PCR. (G) TNPO1 expression in EC109 and KYSE150 cells after transfection with sh-circBCAR3 and pcDNA3.1-circBCAR3 was assessed by PCR. (H) Relative expression of miR-27a-3p and TNPO1 in EC109 cells after transfection with sh-QKI 1/2#. (I) Relative expression of circBCAR3, miR-27a-3p, and TNPO1 in EC109 cells after transfection with sh-E2F7 1/2#. * p < 0.05, ** p < 0.01, *** p < 0.001. [file 12943_2022_1615_MOESM1_ESM.tif]
